# Investigating the impacts of climate and land use/cover changes on the Oueme Delta hydrosystem in Benin, West Africa

**DOI:** 10.1038/s41598-026-39679-x

**Published:** 2026-02-12

**Authors:** René Bodjrènou, Luc Ollivier Sintondji, Marilyn Karen Soudé, Françoise Comandan

**Affiliations:** 1 Institute of Engineering and Management, University of Grenoble Alpes, CNRS, IRD, IGE, Grenoble, France; 2 Hydraulics and Water Management Laboratory, University of Abomey-Calavi, Abomey Calavi, Benin; 3 African Center of Excellence for Water and Sanitation, Abomey Calavi, Benin

**Keywords:** ParFlow-CLM, Water resources, Evapotranspiration (ET), Soil water content (SWC), Water table depth (WTD), Surface runoff (SRO), Climate sciences, Environmental sciences, Hydrology, Geomorphology

## Abstract

Hydrological modeling in deltaic regions remains challenging. This study assesses the impacts of climate change (CC) and land use/land cover change (LULC) on the Oueme Delta hydrosystem using the physics-based integrated model ParFlow-CLM. Surface runoff (SRO), evapotranspiration (ET), water table depth (WTD), and soil water content (SWC) were simulated and evaluated against ERA5 data using performance metrics such as correlation and Kling-Gupta efficiency (KGE). For historical simulations (1975, 2000, and 2013), land-use maps from the West Africa LULC Dynamics project and climate data from WFDE5 were employed. Future projections (2030, 2050, and 2085) relied on climate inputs from CMIP6 datasets, while LULC maps were extrapolated using a Markov chain approach. The model demonstrated strong performance in simulating key components of the water balance, particularly ET (daily scale: Correlation > 0.8; KGE > 0.6). Under constant climate conditions, a 20% reduction in forest cover between 1975 and 2013 showed a negligible impact on water resources. In contrast, CC exerted a substantial influence on the hydrological cycle: increased precipitation led to substantial rises in SRO and ET. Scenario-based projections indicate that LULC changes may amplify climate impacts. Specifically, a precipitation increase exceeding 50% combined with full reforestation could double SRO and increase flood risks. Conversely, a 50% decrease in precipitation coupled with complete deforestation could induce severe ecosystem water stress, reducing SWC by 3.9%. These findings highlight the need for integrated land and water management strategies and inform the development of effective policies for water resource conservation in the context of CC and LULC changes.

## Introduction

West Africa has undergone significant climate change (CC), particularly marked by severe precipitation deficits during the latter half of the twentieth century. Simultaneously, the region has experienced substantial land use and land cover change (LULC), especially through the conversion of forested areas to croplands^1^. Each of these factors—CC and LULC—has individually influenced water resources, primarily by altering the water balance. Ekolu et al.^2^ emphasized that the variability of water resources in sub-Saharan Africa is closely linked to the spatio-temporal variability of precipitation, with climate variability explaining between 30 and 90% of flood occurrence. In parallel, Arfasa et al.^3^ reported that changes in LULC significantly impact water resources, noting that in the Sahel region, runoff coefficients tend to increase as vegetation cover declines. Bagré et al.^4^ similarly observed that the regression of savannahs (− 79.69%) in favor of settlements and bare soils (+ 388.92%) and croplands (+ 79.92%) had a substantial influence on runoff patterns. However, several studies argue that analyzing CC or LULC in isolation may not adequately explain hydrological changes. For instance, Favreau et al.^5^ reported an increase in surface runoff (SRO) and water table depth (WTD) by 4 m despite a 23% reduction in rainfall—attributed to land clearing and changes in soil properties^6^. Similarly, in the Sudanian zone, Vissin^7^ observed reductions in SRO (72% and 66%) in response to only 12–15% rainfall deficits. Bodjrènou and Comandan^1^ also noted that deforestation up to 50% in the Niger basin produced no significant change in SRO. These contrasting results underscore the necessity of jointly analyzing both CC and LULC to better understand their combined impacts on water balance dynamics.

In Benin, numerous studies confirm the ongoing shifts in climate and LULC across different ecological zones^1,8^. Temperatures have risen by approximately 0.20 °C per decade in all three climatic zones^8^, while forest areas have been increasingly converted to agricultural and residential land uses. These trends are expected to intensify in the future^1,9^. Although the hydrological implications of these changes on surface and groundwater have been explored, the impact on soil water content (SWC) remains under-investigated—despite its crucial role in agricultural planning, especially regarding sowing periods and irrigation scheduling. In the Oueme basin, Dossou et al.^10^ reported increasing evapotranspiration (ET), while Lawin et al.^11^ projected increased discharge under the RCP8.5 scenario. Conversely, Biao^12^ suggested that mean annual river discharge could decrease by 15–39% depending on the model and scenario. Cornelissen et al.^13^, focusing on Térou, reported a decrease in runoff by 28–34% and an increase in ET by 5–15%. In the Kandi basin, Kpégli et al.^14^ found surface runoff occurring even during the dry season, likely due to groundwater exfiltration.

Notably, most of these studies have focused on northern and central Benin, regions that share characteristics with the Sahel. However, the sub-equatorial zone, including the Oueme Delta, has received far less attention. This region is currently experiencing rapid population growth, which amplifies pressure on both land and water resources for domestic, agricultural, and energy uses^15^. Hydrological simulations in the delta are constrained by limited availability of hydrometeorological data for calibration and validation^8,16,17^. Bossa et al.^18^ highlight that frequent flooding impedes the installation of measurement stations in the area^16,17^. Moreover, natural resources in the Oueme Delta are undergoing rapid degradation^1^, even though the region is critical to the economy and ecology of southern Benin. It provides fertile agricultural land (from floodplain sediments), supports fisheries and biodiversity, and plays a key role in urban resilience^15,19^. Despite its importance, the hydrological functioning of the Oueme Delta remains poorly understood, particularly during the rainy season when it receives and channels much of the country’s surface water. Understanding this system is essential for the development of Integrated Water Resources Management (IWRM) strategies and the prevention of downstream ecological degradation, especially in lagoon ecosystems.

Furthermore, existing research in the Delta primarily focuses on flood risks and surface water availability^18^. However, not all parts of the delta are flood-prone, making it challenging to assess the actual water availability for different uses. The influence of CC and LULC on groundwater in this sub-equatorial zone is also insufficiently documented. This study is therefore necessary to support integrated management of surface and groundwater resources in the Oueme Delta. It aims to address growing and competing demands across sectors and ensure sustainable access to water in the context of ongoing environmental change. Specifically, this study seeks to assess the current influence of CC and LULC on water resources in the Oueme Delta (i), analyze potential future impacts under different combined scenarios (ii), and explore virtual scenarios to assess the sensitivity of the hydrosystem to extreme changes (iii).

## Materials and methods

### Study area

The Oueme Delta, located in southern Benin, covers an area of approximately 9,000 km^2^, representing about 18% of the Oueme Basin area (52,000 km^2^) and 7.8% of Benin’s national territory. The delta extends between longitudes 2°14.4’ and 2°42.6’, and latitudes 6°19.2’ and 7°19.2’, and is bounded to the south by the Atlantic Ocean. It spans five administrative departments, notably including the Atlantic Department, which hosts Cotonou, Benin’s economic capital, and the Oueme Department, where Porto-Novo, the administrative capital, is located. Population density in the Oueme Delta is particularly high, accounting for 32% of Benin’s total population. This proportion is more than three times the national average, underscoring the region’s demographic and socio-economic importance^20^.

The hydrological regime of the Oueme River within the delta exhibits marked spatial variability, primarily due to soil heterogeneity. In the northern reaches, the river crosses an expansive granite-gneiss peneplain, characterized by low permeability. Further south, it enters sedimentary formations typical of the lower Benin region before flowing through recent alluvial deposits. These deposits, which border the river channel on both sides, contribute to complex hydrodynamic processes^21^.

Climatically, the Oueme Delta benefits from two rainy seasons, with an annual precipitation of approximately 1,300 mm. The mean annual temperature is about 27.5 °C and is projected to increase significantly by the end of the century^18,22^.

Topographically, the southern part of the delta is characterized by very gentle or nearly flat slopes, promoting water dispersion through a dense network of inundation channels. These channels are hydraulically connected to Lake Nokoue and the Porto-Novo Lagoon, forming an intricate wetland system^18^. A key feature of this area is the shallow water table, which remains accessible even during the dry season. This makes the groundwater a vital resource for domestic use and agricultural activities.

**Fig. 1:**
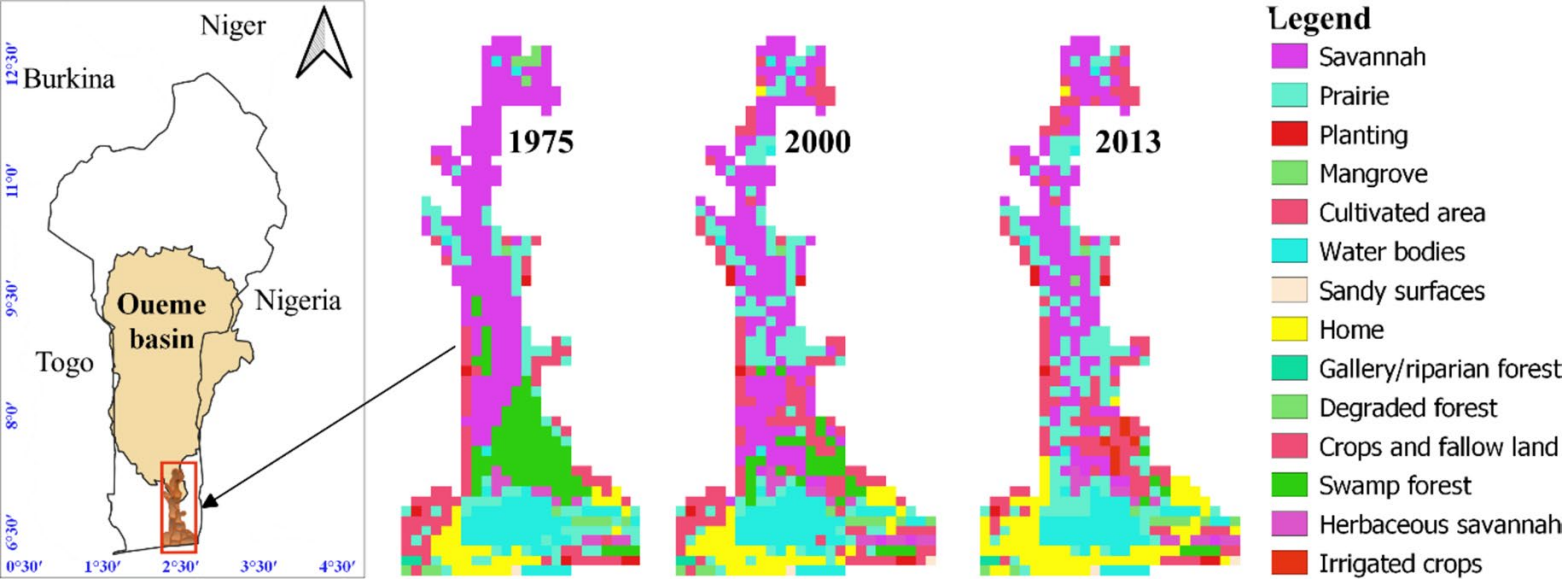
Location of study area.

Regarding land use and cover dynamics, the Oueme Delta has experienced profound transformations over the past decades. According to Bodjrènou et al.^1^, residential areas nearly tripled between 1975 and 2013. At the same time, agricultural expansion has increasingly occurred at the expense of forested areas, as local populations intensify farming and settlement activities within the delta (Fig. 1).

### Hydrological model

The modeling framework employed is ParFlow-CLM version 3.6 (https://parflow.org/), an integrated hydrological model designed to simulate the coupled transport of water and energy across the subsurface, land surface, and atmosphere^23^. It is a physically based, fully distributed model that simulates all components of the water balance, but requires several forcing datasets, especially meteorological, topographic, soil, hydrogeological, and land-use data, as well as initial and boundary conditions.

ParFlow-CLM has been widely applied in West Africa to simulate key components of the hydrological cycle, including surface runoff (SRO), soil water content (SWC), water table depth (WTD), and evapotranspiration (ET). Richard^24^ showed that the model accurately reproduces quasi-saturation conditions (saturation > 95%) in the subsurface during the rainy season. In the Oueme Basin, ^Bodjrènouetal.8,9^ evaluated the model’s performance and found that it successfully reproduced the main water balance components, with Kling–Gupta Efficiency (KGE) values exceeding 0.5, indicating satisfactory model performance. Further validation was provided by Hector et al.^25^, who demonstrated that ParFlow-CLM effectively simulates soil moisture, groundwater fluctuations, streamflow, and evaporation, with KGE values sometimes exceeding 0.8, reflecting strong agreement with observations. Moreover, Herzog et al.^26^ investigated the model’s parametric sensitivity and highlighted the dominant role of near-surface hydraulic conductivity (Ks) and clay content in controlling streamflow generation in the West African critical zone, showing that streamflow is more sensitive to shallow regolith properties than to deeper subsurface characteristics.

### Data description and sources

#### Land use and land cover (LULC) data

Hydrological modeling in this study was driven by multiple datasets with high spatial and temporal resolution. Land use and land cover (LULC) data were obtained from the Land Use and Land Cover Dynamics (LULCD) project, which provides spatially consistent datasets at a 2 km × 2 km resolution for three key reference years: 1975, 2000, and 2013^27^.

These LULC maps were produced using a combination of satellite imagery and field surveys and constitute essential inputs for simulating the temporal evolution of surface conditions in the Ouémé Delta. The selected reference years correspond, respectively, to periods characterized by high humidity, intensified agricultural mechanization, and increased rainfall, thereby capturing major phases of land use transformation in the study area. The LULCD datasets are openly accessible via the USGS platform at: https://www.usgs.gov/centers/eros/science/land-use-and-land-cover-trends-west-africa.

Future LULC projections were generated using the Markov Chain algorithm implemented in the Land Change Modeler (LCM) module of IDRISI TerrSet 2020 software (https://clarklabs.org/terrset/). This approach estimates future land cover distributions by extrapolating historical transition probabilities and is particularly suitable for analyzing land use dynamics in complex socio-environmental systems. Although land use projections inherently involve uncertainties, these uncertainties are considered negligible in the present study, given the strong agreement between simulated land cover areas and observed data reported by Bodjrènou et al.^1^.

#### Climate data

Historical climate forcing was derived primarily from the WFDE5 dataset, a bias-adjusted global reanalysis product developed from ECMWF’s fifth-generation reanalysis (ERA5). WFDE5 provides consistent global coverage from 1979 to 2019 and is widely recognized for its suitability in hydrological modeling applications^28^.

For more periods not covered by WFDE5, ERA5 reanalysis data were used to complement the historical climate forcing. ERA5 is known for its strong ability to represent meteorological variability across the diverse climatic zones of Benin^16,29^. ERA5 data have a spatial resolution of 0.25° × 0.25°, which is finer than that of WFDE5 (0.5° × 0.5°), with both datasets available at an hourly temporal resolution.

Future hydrological simulations were driven by climate projections from CMIP6 models, with temperature and precipitation data extracted from the CNRM-CM6-1 and IPSL-CM6A-LR models, respectively, under the SSP1-2.6 (SSP126) scenario. The CNRM-CM6-1 model was developed by the “Centre Européen de Recherche et de Formation Avancée en Calcul Scientifique” (CERFACS) and provides outputs at a spatial resolution of 1.4° × 1.4° with a daily temporal resolution^30^. The IPSL-CM6A-LR model, developed by the Institut Pierre-Simon Laplace (IPSL), has a spatial resolution of 2.5° × 1.3°, also at a daily time scale^31^.

All climate projection data were freely downloaded from the Copernicus Climate Data Store (https://cds.climate.copernicus.eu/datasets). Both models were previously evaluated and found suitable for representing the regional climate of southern Benin, including the Ouémé Delta, as reported by Bodjrènou et al.^22^. Prior to their use in hydrological simulations, the climate model outputs were bias-adjusted using a combined Quantile Mapping (QM) and delta change approach, following the methodology detailed in Bodjrènou et al.^1,22^.

#### Validation data

The data used to evaluate the model’s performance were obtained from the Copernicus Climate Data Store (https://cds.climate.copernicus.eu/datasets). Specifically, this study employed the fifth-generation atmospheric reanalysis produced by the European Centre for Medium-Range Weather Forecasts (ECMWF), known as ERA5, which is available at a spatial resolution of 0.25° × 0.25° and an hourly temporal resolution.

The reanalysis datasets were used without any transformation or post-processing for surface runoff (SRO), soil water content, evapotranspiration (ET), or water table depth/deficit (WTD), thereby ensuring an independent and unbiased evaluation of the model’s predictive capability. ERA5 data were downloaded for the period 1995–2005, corresponding to the model validation period.

### Forcing time series

#### LULC forcing time series

To harmonize the land cover input for the hydrological model, the original LULCD land use maps were reclassified into four aggregated classes: high vegetation, low vegetation, urban, and water. The high vegetation class grouped forested and wooded formations (e.g., forest, savannah, plantation, gallery forest, degraded forest, woodland, and flooded forest), while the low vegetation class included various agricultural and herbaceous land uses (e.g., wetlands, steppes, irrigated and non-irrigated cropland, grassy savannah, and palm-dominated areas). The urban class encompassed bare soil, urban areas, sand, and rock surfaces, whereas the water class included permanent and seasonal water bodies. To capture vegetation dynamics, Leaf Area Index (LAI) time series from the ERA5 reanalysis were used, distinguishing between high and low vegetation types based on consistent classification criteria. High vegetation LAI covered evergreen, deciduous, and mixed forests—consistent with our reclassified maps—while low vegetation LAI included grasslands, crops, marshes, and other low-cover systems. These LAI time series, derived from satellite observations over 2000–2008, align with the model’s simulation period (1995–2005) and ensure realistic representation of vegetation dynamics over time. The proportional distribution of each land cover class for the base years and future scenarios is summarized in Table 1. LAI values for each land cover category were computed using a standard aggregation formula integrating temporal and spatial fractions.

**Table1:** Percentage of each land-use class.

CLM class	1975	2000	2013	2030	2050	2085	SEN1	SEN2
Forest (8)	49,8	38,2	27,3	26,6	15,0	0	0	100
Water (17)	13,5	13,8	12,4	14,1	14,4	14,88	0	0
Residential (13)	6,7	8,7	14,7	10,7	12,7	15,9	0	0
Agriculture (19)	30,0	39,3	45,6	48,6	57,9	69,22	100	0
Total	100	100	100	100	100	100	100	100

1 $${LAI}_{h,mod}= {LAI}_{h,era}*{P}_{h,era}$$

With ${LAI}_{h,mod}$= Leaf area index, of h vegetation (low or high) used in the model, ${LAI}_{h,era}$= Leaf area index, of h vegetation (low or high) in ERA5, ${P}_{h,era}$= percentage, of h vegetation (low or high) in ERA5.

#### Meteorological forcing time series

**Fig. 2:**
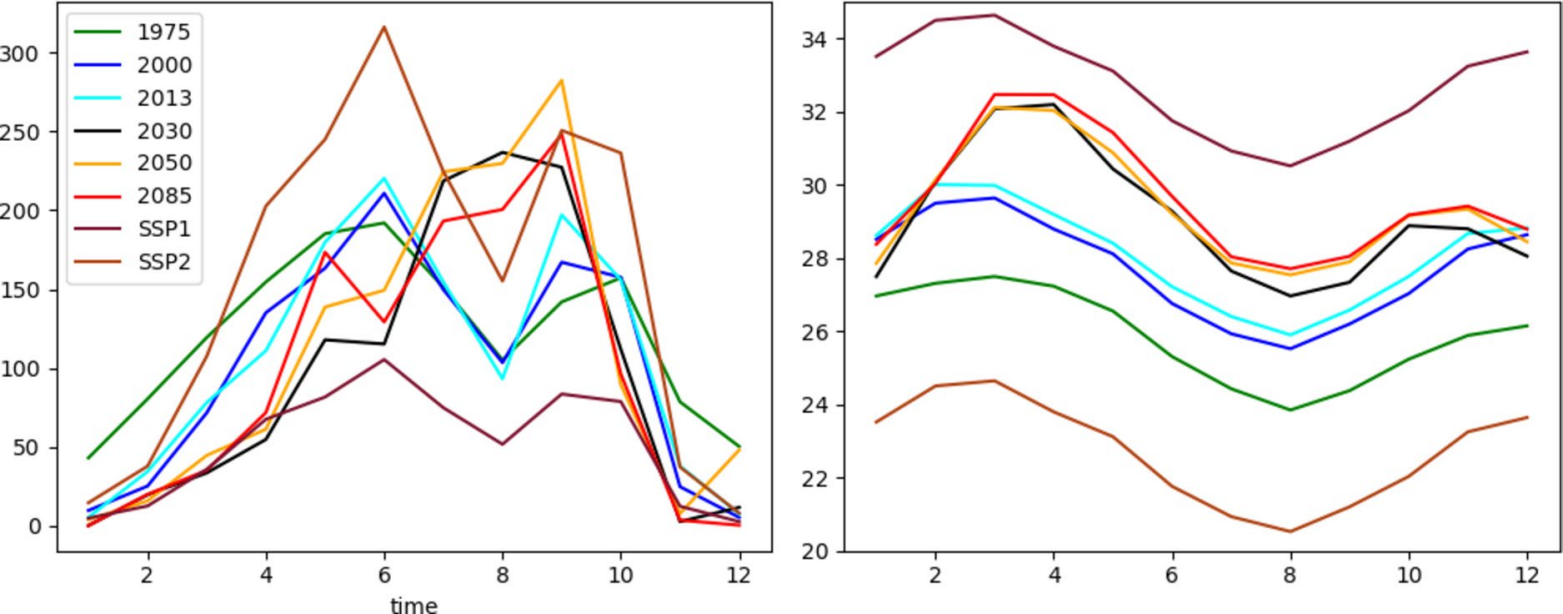
Forcing precipitation (left) and temperature (right).

Meteorological forcing for the model simulations was derived using eleven-year climate data windows, centered on the reference year of each land use map. This approach ensures that each simulation reflects consistent climatic conditions associated with corresponding land cover states. For instance, the land use map from the year 2000 was paired with climate data spanning 1995 to 2005. For scenario-based simulations, two virtual climate configurations were introduced to evaluate extreme changes: the first (SSP1) assumes a 5 °C increase in mean temperature accompanied by a 50% decrease in precipitation, while the second (SSP2) posits a 5 °C temperature decrease and a 50% increase in precipitation. These synthetic scenarios are designed to explore how systems respond to contrasting climate stress conditions. This enables the assessment of the Oueme Delta’s hydrological resilience and sensitivity. Figure 2 illustrates the average annual cycles of precipitation and temperature across the simulations, while Table 2 summarizes the mean climatic values for each simulation period.

**Table 2:** Average annual temperature and precipitation for each simulation period.

Variable	1975	2000	2013	2030	2050	2085	SEN1	SEN2
Precipitation (mm)	1459	1224	1275	1150	1295	1172	612	1835
Temperature (°C)	26.3	27.7	28.1	29.1	29.4	29.6	32.7	22.7

### Evaluation criteria of model

The 2000 simulations, based on land use data from the same year, were evaluated against the ERA5 reanalysis outputs for surface runoff (SRO), soil water content (SWC), evapotranspiration (ET), and water table depth (WTD) to assess the model’s ability to reproduce hydrological processes in the Oueme Delta. The comparison criteria were based on correlation (Eq. 2), root mean square error (Eq. 3) and Kling Gupta efficiency^32^, which aggregates other evaluation criteria, in particular the mean ratio and the coefficient of variation ratio (Eq. 4). In addition, water availability in the hydrosystem is estimated by subtracting water losses due to evapotranspiration from rainfall contributions. Runoff water is considered useful for the population, as it is blocked downstream by the Atlantic Ocean (Fig. 1).

2 $$c=\frac{\sum_{i=1}^{n}\left({Y}_{i}-\overline{Y }\right).\left({X}_{i}-\overline{X }\right)}{\sqrt{\sum_{i=1}^{n}{\left({Y}_{i}-\overline{Y }\right)}^{2}}.\sqrt{.\sum_{i=1}^{n}{\left({X}_{i}-\overline{X }\right)}^{2}}}$$

3 $$\mathrm{RMSE}= \frac{{\sum }_{\mathrm{i}=0}^{\mathrm{n}}\left|{X}_{i}-{Y}_{i}\right|}{{\sum }_{\mathrm{i}=0}^{\mathrm{n}}\overline{{X }_{i}}}$$

4 $$\text{KGE }=1-\sqrt{{\left(\mathrm{c}-1 \right)}^{2}+{\left(\frac{{\sigma }_{rea}}{{\sigma }_{obs}}-1 \right)}^{2}+{\left(\frac{\overline{Y} }{\overline{X} }-1\right)}^{2}}$$

where:${X}_{\mathrm{i}}$ is the observation data,${\mathrm{Y}}_{\mathrm{i}}$ is the reanalysis data and n is the sample size. ${\sigma }_{obs}$ and ${\sigma }_{rea}$ for standard deviation of observational and reanalyses data, respectively.

### Methodological summary and experimental protocol

**Fig. 3:**
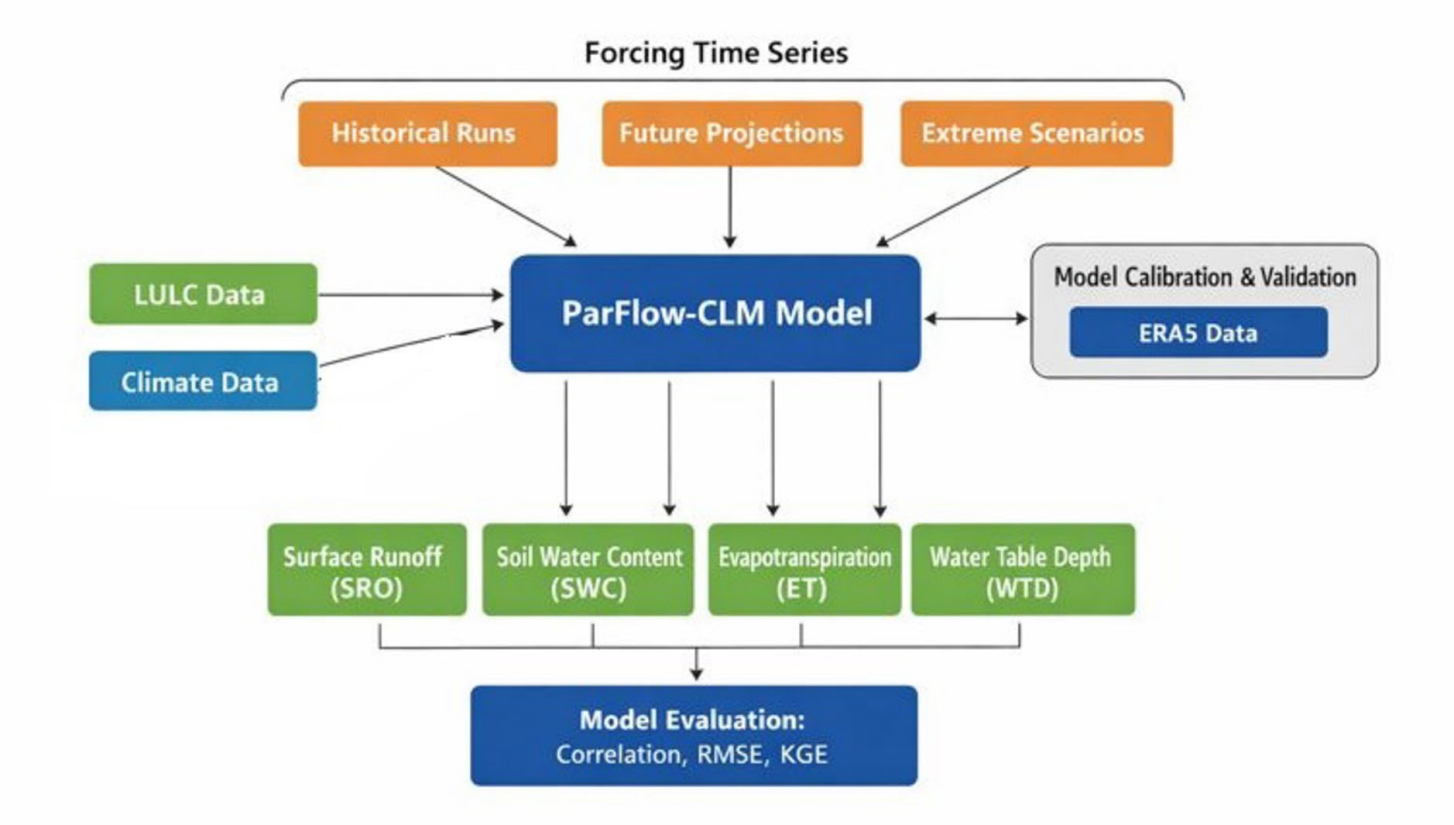
Experimental Workflow of ParFlow-CLM Simulations in the Ouémé Delta.

This study employed a structured hydrological modeling framework to assess the impacts of climate change (CC) and land use/land cover (LULC) dynamics on the Oueme Delta (Fig. 3). The modeling approach integrates the physical, topographical, geomorphological, and climatic characteristics of the delta to ensure a realistic representation of its hydrological processes.

Hydrological simulations were performed at the daily scale using the ParFlow-CLM model, based on a two-dimensional (2D) hillslope representation, to explore both current and projected future scenarios. Three primary modeling scenarios were defined to disentangle the individual and interactive effects of climate and land use changes:

- Climate-Only Scenario: LULC was held constant while climate inputs were varied, isolating the impacts of climate change on water resources.
- LULC-Only Scenario: Climate data were kept fixed to evaluate the exclusive effects of land use and cover dynamics.
- Combined Scenario: Both climate and LULC inputs were varied simultaneously to assess their interactive and cumulative effects on key hydrological variables.

Model outputs included surface runoff, infiltration, evapotranspiration, and soil water availability, providing insights into water partitioning relevant for agriculture, groundwater recharge, and ecosystem health under evolving environmental conditions.

These simulations addressed two main objectives: (1) quantifying the influence of historical and current climate and land cover changes on water resources, and (2) assessing potential future alterations in hydrological processes under projected scenarios. To explore extreme or hypothetical conditions, additional virtual scenarios were developed by combining synthetic climate variations (± 50% precipitation and ± 5 °C temperature) with contrasting LULC assumptions, such as complete deforestation versus full reforestation. This framework enables a robust evaluation of the resilience and vulnerability of the Oueme Delta’s hydrological system under multiple environmental stressors.

## Results

### Model performance in simulations

**Fig. 4:**
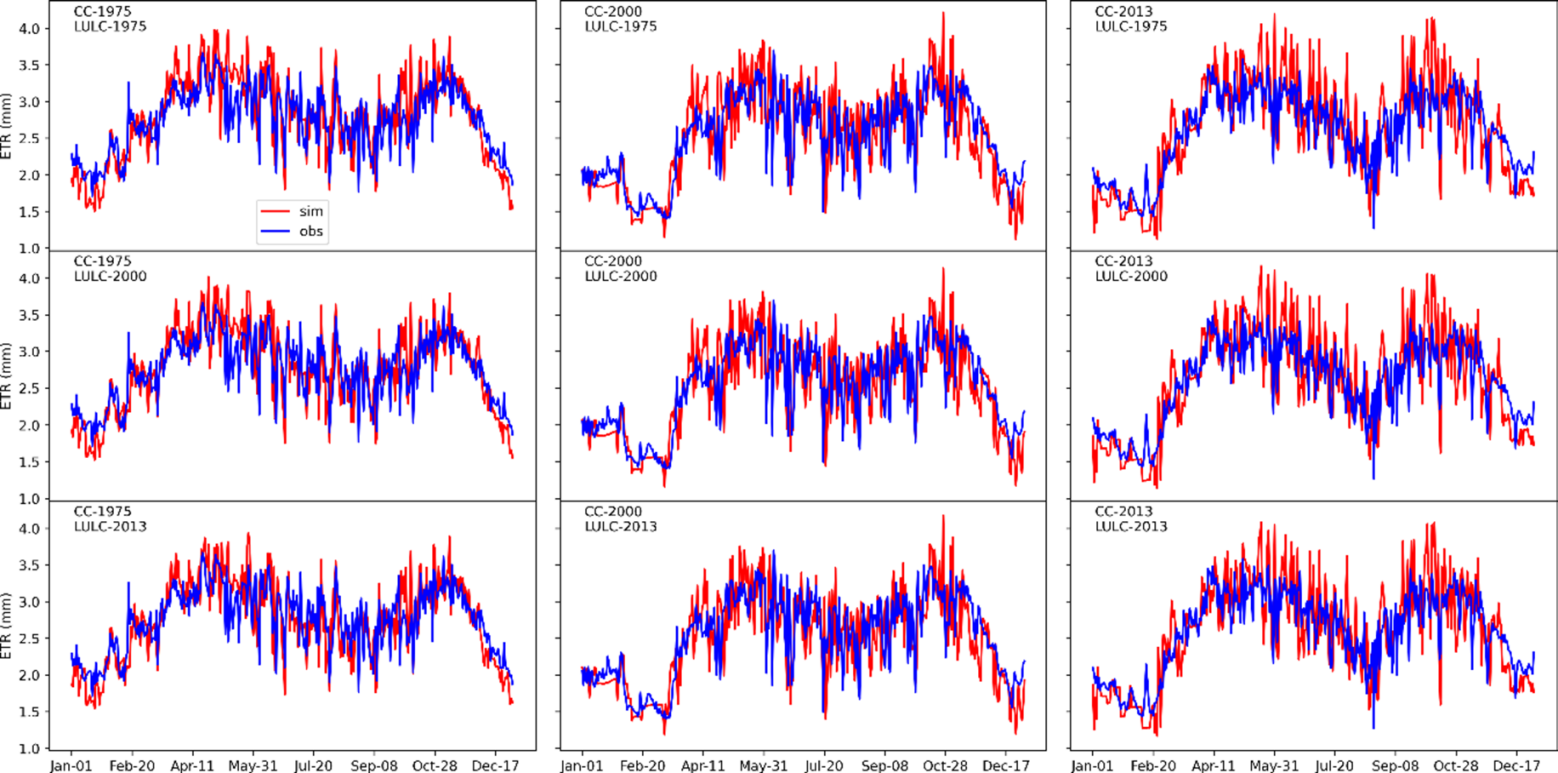
Simulated (red) and observed (blue) evapotranspiration in the Oueme Delta under different land use and land cover (LULC) conditions.

Figure 4 illustrates the impact of recent land use and land cover (LULC) changes on evapotranspiration (ET) in the Oueme Delta, while Table 3 presents the performance metrics (correlation, RMSE, and KGE) of simulated variables compared to ERA5 reanalysis data at the daily scale. The ET exhibits a bimodal seasonal cycle, with peaks between April–May and again in October, corresponding to the region’s two rainy seasons. Conversely, the lowest ET occurs from November to February, aligning with the long dry season in southern Benin. This seasonal pattern highlights a strong interaction between ET and precipitation, as greater water availability leads to higher evapotranspiration.

**Table 3:** Correlation performance (corr), KGE and RMSE for SRO, ET, SWC, WTD.

Metrics	Year	ET			SRO			SWC			WTD		
		LU1	LU2	LU3	LU1	LU2	LU3	LU1	LU2	LU3	LU1	LU2	LU3
Corr	1975	0.81	0.81	0.81	0.44	0.44	0.44	0.77	0.77	0.77	0.12	0.12	0.13
	2000	0.78	0.78	0.77	0.30	0.31	0.31	0.90	0.91	0.90	-0.67	-0.67	-0.68
	2013	0.79	0.79	0.79	0.20	0.19	0.20	0.79	0.80	0.79	-0.51	-0.51	-0.51
KGE	1975	0.64	0.68	0.68	0.34	0.31	0.33	0.10	0.10	0.10	-0.03	-0.03	-0.03
	2000	0.72	0.75	0.74	0.29	0.31	0.31	0.10	0.12	0.10	-0.77	-0.77	-0.77
	2013	0.62	0.64	0.67	0.19	0.18	0.19	0.07	0.07	0.08	-0.63	-0.63	-0.62
RMSE	1975	0.34	0.32	0.32	4.07	4.08	4.09	6.53	6.52	6.53	8.24	8.24	8.22
	2000	0.41	0.40	0.40	4.97	4.98	5.04	6.46	6.45	6.46	11.72	11.81	11.87
	2013	0.44	0.43	0.42	3.69	3.65	3.65	6.57	6.55	6.54	9.79	9.86	9.93

Note: LU1, LU2, and LU3 represent the LULC maps for 1975, 2000, and 2013, respectively.

Model performance for ET is generally strong, with KGE values exceeding 0.7, confirming the model’s robustness in reproducing observed ET patterns. The highest ET performance (KGE = 0.75) was recorded when using 2000 climate data alongside the 2000 land use map, slightly outperforming the 1975 (KGE = 0.72) and 2013 (KGE = 0.74) land use maps. Similar patterns are observed for surface runoff (SRO) and soil water content (SWC), suggesting improved model accuracy when climate and land use inputs are chronologically consistent. However, simulations using 1975 data performed slightly worse, likely due to the reduced reliability of climate reanalysis products for earlier periods^8^. In contrast, water table depth (WTD) simulations showed poor agreement with observations, with negative correlations and KGE values, probably reflecting limitations in the validation dataset for this variable.

### Current impacts of climate change on water resources

**Fig. 5:**
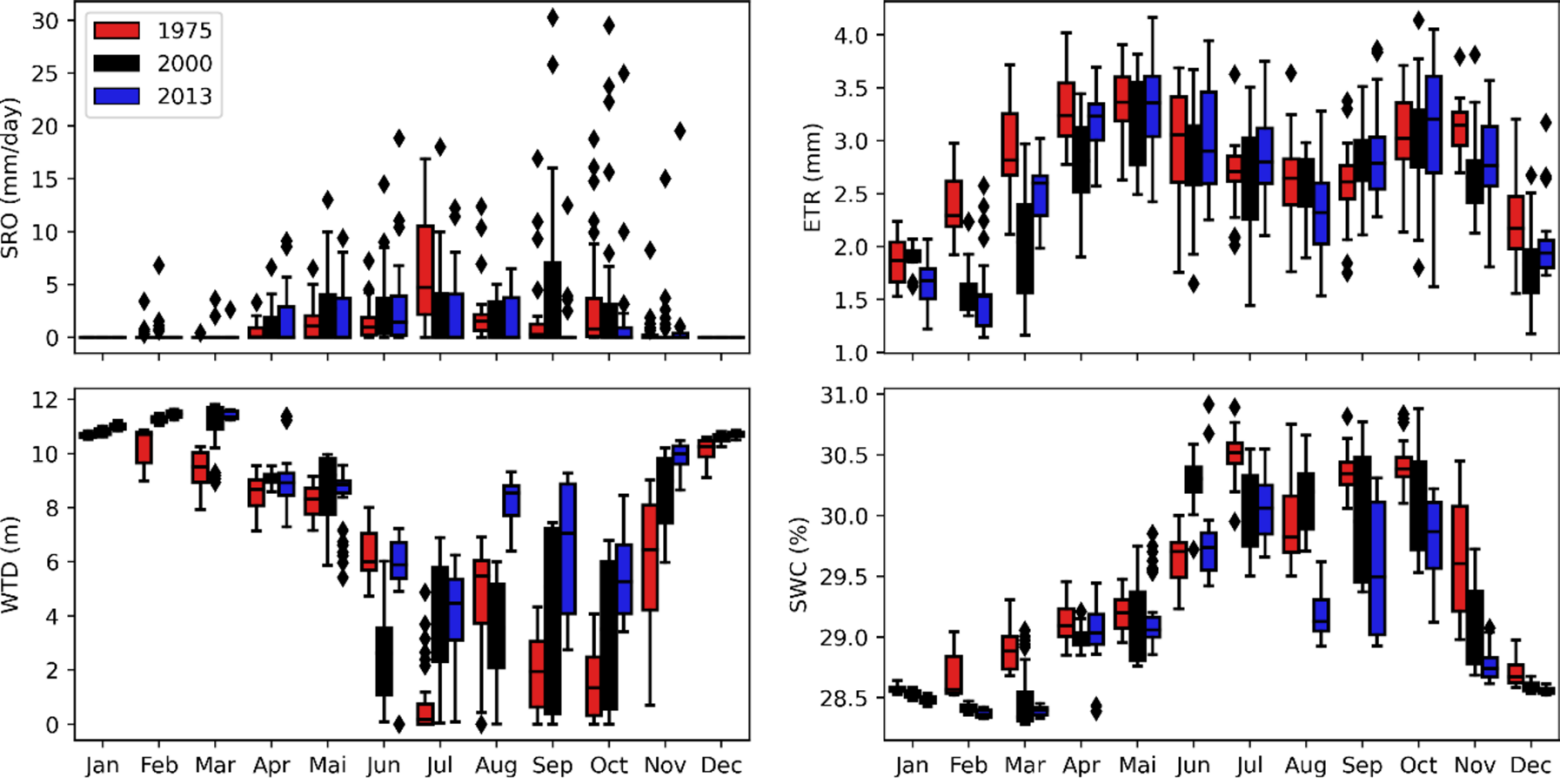
Impact of climate change on water balance terms. Red shows simulations with the 1975 land-use map, black shows simulations with the 2000 land-use map and blue shows simulations with the 2013 land-use map.

Figure 5 compares the 1975, 2000, and 2013 simulations to highlight the influence of CCs on all water balance terms. Note that only the 2000 land use map was used in this case. The average values for each month are reported in Table 4. Analysis of Fig. 5 shows that the simulated variables are very consistent with observations. The lowest flows are noted between December and March, which coincides with the long dry season in the delta. Consistency with the climate is also demonstrated in June, where the simulated SRO is moderately higher in 1975 than in 2000 and 2013. In this month, we note 43.2 mm/d, 83.7 mm/d and 90.6 mm/d for the 1975, 2000 and 2013 SRO, respectively. This variation follows the same trend as the variation of precipitation (highest to lowest) and temperature (lowest to highest) between 1975 and 2013 in this month. The situation is similar for other months and/or variables, such as WTD, which approaches 0 m in July 1975, but has decreased in other periods, demonstrating the significant effect of CCs. However, there are also some inconsistencies and even contradictory answers. As an example, we can mention that the SRO for the 1975 simulation shows its maximum peak in July, while the maximum precipitation is recorded in June. These inconsistencies can be explained by the wide distribution of precipitation within the same month, the influence of other climatic variables used as inputs to the model, or even the response time of the basin^6,33^.

**Table 4:** Mean value of each parameter.

Variable	Year	Jan	Feb	Mar	Apr	May	Jun	Jul	Aug	Sep	Oct	Nov	Dec
SRO	1975	0.0	5.3	0.4	16.9	47.3	43.2	197.5	65.5	54.5	106.8	14.6	0.0
	2000	0.0	11.4	5.6	33.7	97.5	83.7	85.3	61.2	145.2	125.1	25.2	0.0
	2013	0.0	0.0	2.6	54.5	65.5	90.6	68.4	52.5	22.4	51.3	24.7	0.0
ET	1975	57.6	67.1	90.0	99.1	104.4	88.9	83.3	81.6	77.9	94.1	93.5	69.4
	2000	59.2	47.1	61.9	84.2	99.3	84.2	81.7	79.0	84.1	93.3	80.6	57.1
	2013	50.9	43.2	78.2	95.9	103.7	90.0	88.0	72.5	85.8	97.3	84.1	62.3
WTD	1975	10.7	10.3	9.4	8.5	8.2	6.3	0.8	4.7	1.9	1.5	6.0	10.1
	2000	10.8	11.3	11.1	9.1	8.6	2.5	3.8	3.4	4.0	3.4	8.6	10.6
	2013	11.0	11.4	11.4	8.9	8.4	5.7	4.0	8.2	6.5	5.5	9.9	10.7
SWC	1975	28.6	28.7	28.9	29.1	29.2	29.6	30.5	29.9	30.4	30.4	29.7	28.7
	2000	28.5	28.4	28.5	29.0	29.1	30.3	30.1	30.1	30.0	30.1	29.1	28.6
	2013	28.5	28.4	28.4	29.0	29.2	29.8	30.1	29.2	29.6	29.8	28.8	28.6

### Current impacts of LULC changes on water resources

**Fig. 6:**
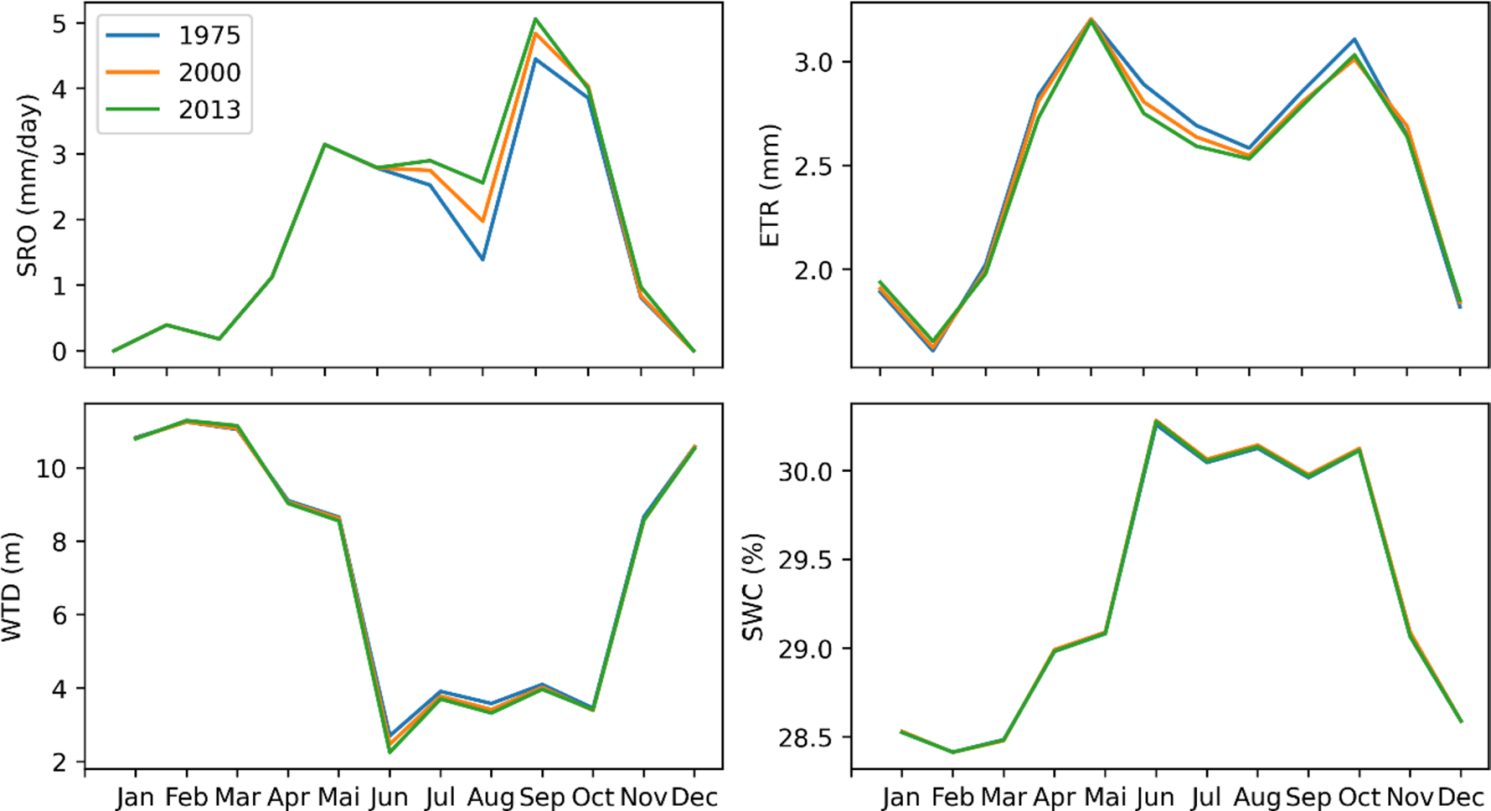
Impact of changes in surface conditions on water balance terms.

Figure 6 shows the impact of LULC changes on the water balance terms. Analysis of this figure shows that land use changes have a significant impact on the SRO in July–August-September (JAS). During this period, we can see that the 1975 simulations generate a larger SRO than the 2000 simulation, and the latter generates a larger SRO than the 2013 simulation. For the other months, there is no significant difference is observed between the SRO simulations in 1975, 2000 and 2013. The annual averages of the three simulations are almost identical (no significant difference), which means that a 20% reduction in forest area (77.70% in 1975, vs. 57.10% in 2013) has no significant impact on water resources in the Oueme delta. The situation is almost identical for the WTD. In 1975, the WTD values were lower than in 2000, and these were lower than in 2013. During the JAS period, the third land use map simulates WTD close to zero. This phenomenon is practically real in certain parts of the Oueme delta, such as the area around Lake Nokoué area, located entirely to the south of the basin. Since the phenomenon is more pronounced with the 2013 map than in the other two, we can conclude that changes in land use have a (non-significant) impact on groundwater recharge in the deltaic zone.

In the JAS period, the ET with the 1975 simulations is higher than the simulations based on the 2000 land use map, and the latter is higher than the 2013 simulations. During the dry season, especially in December-January–February, the ET with the 1975 simulations is lower than the simulations based on the 2000 land use map, and the latter is lower than the 2013 simulations. For SWC, there is no significant difference between the 1975, 2000, and 2013 simulations. This can be explained by the fact that changes in the physical properties of the soil are not considered in the model. In reality, changes in LULC can lead to changes in soil properties. For example, the greater the root density, the more porous the soil may be, whereas a soil without roots, which may be less porous and therefore retain more water^34,35^.

### Potential impacts of global changes on water availability

**Fig. 7:**
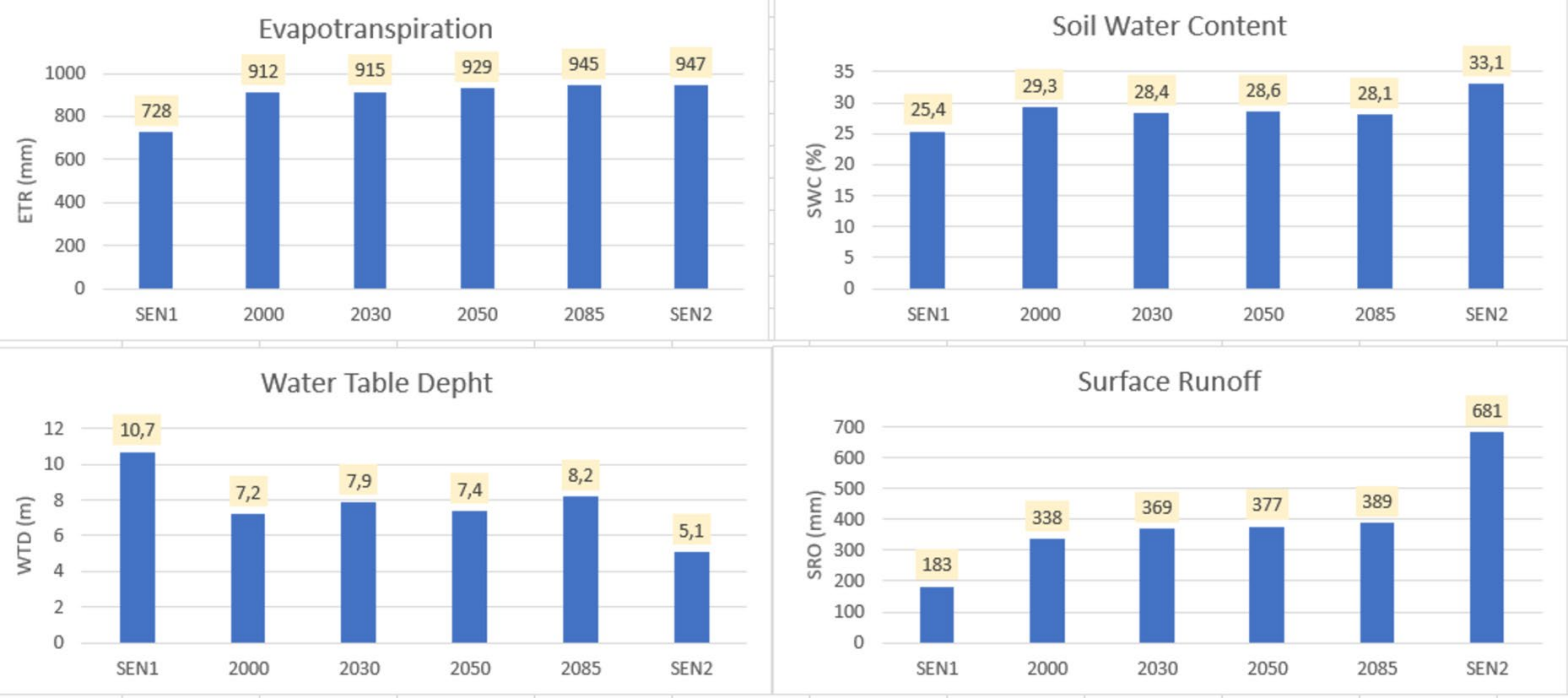
Potential impacts on water balance terms.

Figure 7 shows the potential impacts of global change on ET, SWC, WTD and SRO. The amount of water potentially available for each horizon and scenario is shown in Table 5. Analysis of Fig. 7 shows that the WTD could increase over time, rising from 912 mm in 2000 to 945 mm in 2085, an increase of 3%. For the SRO, there is an increase of 15% between these two sub-periods (338 mm and 389 mm in 2000 and 2085 respectively). The runoff coefficient increased from 27.6% in 2000 to 33.2%, an increase of about 5.6%. Evapotranspiration accounted for 75% of the precipitation in 2000 and 81% in 2085 (Table 5). This high ET value can be explained by the presence of water bodies in this environment. It is an environment that collects water upstream of the Oueme basin and remains wet almost throughout the year^15^. The low flow coefficient compared to other studies can be explained by the low slope adopted in the model.

**Table 5:** Estimated delta water availability (D).

Period	Rainfall (mm)	ET (mm)	D (mm)	D (%)
SEN1	612	728	-116	-19
2000	1224	912	312	25
2030	1150	915	235	20
2050	1296	929	367	28
2085	1172	945	227	19
SEN2	1835	947	888	48

The results show that ET is projected to continue increasing in the Oueme delta despite a reduction in forested areas, likely driven by global warming and rising temperatures. SRO is also expected to increase, probably because runoff generation is more efficient over bare land than over forested surfaces. However, the results do not indicate a proportional relationship between CC and water resources, nor between land use/land cover (LULC) changes and hydrological responses.

Under the SEN1 scenario, which assumes a decrease in land surface conditions and precipitation, indicates that ET would be lower than in the reference year 2000 (728 mm for SEN1 versus 912 mm in 2000). Groundwater levels would be deeper, with the water table depth increasing from 7.2 m in 2000 to 10.7 m under SEN1, corresponding to a deepening of 3.5 m. Surface runoff would decrease markedly from 338 to 183 mm (− 46%), while soil water content (SWC) would decline from 29.3% to 25.4%, representing a reduction of 3.9 percentage points relative to 2000.

In contrast, the SEN2 scenario, which assumes lower temperatures, increased precipitation, and enhanced vegetation cover, exhibits the opposite response (Fig. 7). SWC would increase to 33.1%, corresponding to a gain of 3.8 percentage points relative to 2000. Surface runoff would also rise substantially, reaching 681 mm (more than double the 2000 value), while the water table would become shallower, with depth decreasing to 5.1 m, corresponding to a rise of 2.1 m relative to the reference year (Fig. 7). Finally, the assessment of water availability indicates that the population could experience an annual water deficit of approximately − 116 mm·yr⁻^1^ (Table 5).

## Discussion

The quality of the simulations presented in this study demonstrates the ability of the model to capture hydrological processes in the Oueme Delta. This result corroborates the studies of Richard et al.^24^, which indicate that the Parflow-CLM model performs well in simulating water balance conditions in tropical environments, especially when model parameters are consistent with observational data.

More recently, Bodjrènou et al.^1^ reported that ParFlow performs very well in simulating evapotranspiration. Their KGE performance was similar to that reported in the present study. The previous authors also reported good model performance in simulating other variables, particularly WTD (KGE > 0.7). In contrast, the present study shows weaker performances for WTD, which is likely attributable to the limitations in the ECMWF fifth-generation reanalysis (ERA5) data used for model validation. Indeed, Bodjrènou et al.^22^, who assessed the consistency of ERA5 data with in situ observations, reported an overestimation of approximately 4.73 m in WTD. Furthermore, the quality of forcing data and the methods applied for their processing can substantially affect model simulations. Using land-use maps, Bodjrènou et al.^16,17^ showed that predictions based on the Markov chain approach, although effective, do not fully reproduce land-use classes. Similar limitations apply to meteorological data, which has motivated the widespread application of bias-correction techniques prior to their use in hydrological modeling^1,28^.

The analysis of the impact of CC, assuming a constant surface condition, shows significant effects on water resources. The lowest flows were observed in the dry season and the highest in the rainy season. These results corroborate several studies conducted in the sub-region. Arfasa et al.^3^ mention that rising temperatures have affected all sectors, and reduced rainfall will reduce river flows and increase evaporation due to a drier atmosphere. The WTD is almost at the surface during the rainy season for the 1975 simulations. It has decreased over time, with significant differences in almost every month. This is certainly what led Arfasa et al.^3^ to conclude that CC will reduce the amount of water available in reservoirs for irrigation. The significant difference in almost every month between the 1975, 2000 and 2013 simulations also confirms that water resources in the Oueme Delta are highly vulnerable to CC^36^.

The results also show that under a constant climate, the balance terms WTD and SWC in particular are not very sensitive to changes in LULC in the dry season. These results contradict the study of Op de Hipt et al.^37^, who report that changes in LULC led to an increase in water yield efficiency (WYE) between 3.6% and 46.5% in Burkina-Faso. In the same vein, Le Lay (2008) indicates that the Bétérou basin has a hydrological response time six times longer than the other basins. This justifies the different response of the Oueme delta compared to the upper Oueme basin. Non-significant impacts may also be related to the low sensitivity of the model to vegetation parameters and the fact that soil physical properties remain unchanged. According to Bagré et al.^4^, streamflow trends are related to the dynamics of land use in the region. However, the effect of precipitation can be offset out by that of soil permeability^6^. Similarly, agricultural land provides higher groundwater recharge than other land uses for all soil hydrological conditions^38^. These elements demonstrate the need to modify soil physical properties with LULC changes in the model. Another reason is that groundwater recharge depends not only on the physiographic characteristics of the watershed, but also on other practices that occur there^39^. The low impact of LULC changes in this study may also be the reason for the dominance of the forest zone: savanna still occupies more than 50% between 1975 and 2013 (Bodjrènou et al., 2023S).

The assessment of potential impacts clearly showed that the two factors, CC and LULC changes, together have significant impacts on water resources in the Oueme Delta. The impacts are characterized by an increase in ET and SRO, which are unprofitable water outflows for local farmers. The differences are observed in the current and future periods, and are remarkably significant between SEN2 (scenario of increased precipitation and vegetation) and SEN1 (scenario of decreased precipitation and vegetation), which predicts a water deficit. These results are in line with the studies of Höllermann et al.^40^, who claim that water resources will soon be an element of conflict for the population living in the Oueme basin. Höllermann et al.^40^ also found similar results, concluding that water resource withdrawals in the Oueme basin to meet demands in the agricultural, domestic and other sectors will almost triple by 2025. In the same vein, Arfasa et al.^3^ also mention that the current demand for freshwater for irrigation purposes in West Africa will triple by 2050. Studies conducted in Burkina by Op de Hipt et al.^37^ have shown that the combined effect of the two factors on runoff can be placed in the same category as the isolated effect of LULC changes. According to Etène et al.^41^, the combined LULC and CC changes in the Oueme basin at Kaboua have no effect on runoff, but rather on ETP. However, each of these factors has significant effects in Kaboua, Bonou and Donga. In the Sudanese zone, the same authors find that the combined effect of the two factors is similar to that of climate on all the highlighted hydrological balance terms. The impacts presented in our studies are generally not significant on the balance terms, certainly because the effect of CC is offset by the effect of LULC Belemtougri et al.^6^. Moreover, the results seem to contradict what has been observed in the other basins of the country and even in the Sahel zone, leading to the conclusion that the Oueme Delta has a particular hydrological cycle. Séguis et al.^42^ explain the difference by the presence of two aquifers in some parts of the country, compared to one in others. The authors also point out that the second layer is seasonal, shallower and accounts for most of the runoff in the basins^14^. These elements may justify the different response observed in relation to the Oueme Delta.

## Conclusion and recommendations

This study assessed the impacts of climate change (CC) and land use/land cover (LULC) changes on the hydrosystem of the Oueme Delta using the physically based ParFlow-CLM model. The model effectively reproduced major water balance components, with satisfactory performance metrics, particularly when climate and LULC data were temporally aligned. However, discrepancies—especially for variables like water table depth (WTD)—highlight the limitations of reanalysis data for model validation. Additionally, some parameters, such as soil water content (SWC), showed limited sensitivity to LULC changes, likely due to internal model constraints and parameter interdependencies. To improve realism, future modeling efforts should isolate individual variables and be supported by field observations through newly installed gauging stations.

Findings show that climate change exerts a dominant influence on hydrological dynamics in the Oueme Delta, with LULC changes acting as amplifying factors. However, no direct proportional relationship was observed between either CC or LULC and all water resource components, especially during the dry season. Simulations under a pessimistic scenario—50% reduction in rainfall, 5 °C rise in temperature, and complete forest loss—project a severe water deficit in the region. Conversely, a scenario involving increased rainfall and forest recovery suggests a risk of prolonged flooding. These outcomes underline the urgent need for policy interventions, including climate adaptation strategies, reforestation efforts, and upstream water infrastructure (e.g., hydro-agricultural dams) to balance competing risks of scarcity and excess.

Finally, the study highlights the complex and dynamic nature of the Oueme Delta, influenced by both freshwater and seawater inputs, which vary seasonally and alter the ecological and hydrological balance. This underscores the importance of future investigations into freshwater-saltwater interactions, as well as the socio-economic drivers of LULC, particularly land tenure dynamics. In addition, modeling should expand to capture inter-annual variability, thereby informing water management strategies tailored to long-term agricultural resilience and ecosystem stability in the Delta.

## Data Availability

The datasets generated and/or analyzed during the current study are available from the corresponding author upon reasonable request.

## References

[1] Bodjrènou, R. & Comandan, F. Influence of land use dynamics on water resources in Benin (West Africa). *LHB Hydrosci. J.* **109**(1), 1–13. 10.1080/27678490.2023.2233482 (2023).

[2] Ekolu, J.,,Dieppois, B. & Tramblay, Y. Variability in flood frequency in sub-Saharan Africa: The role of large-scale climate modes of variability and their future impacts. *J. Hydrol*. **640**, 131679. 10.1016/j.jhydrol.2024.131679 (2024).

[3] Arfasa, G. F., Owusu-Sekyere, E., Doke, D. A. & Aygei Ampofo, J. Impacts of climate and land use/cover changes on the sustainability of irrigation water in West Africa: A systematic review. *All Earth* **36**(1), 1–13. 10.1080/27669645.2024.2308371 (2024).

[4] Bagré M.P., Yonaba R., Sirima B.A. & Somé Y.S.C. Influence of land use changes on flows in the Massili watershed in Gonsé (Burkina Faso). *Vertigo Rev. Electron. Sci. l'environ*. 10.4000/vertigo.39765 (2023).

[5] Favreau, G. et al. Land clearing, climate variability, and water resources increase in semiarid southwest Niger: A review. *Water Resour. Res. Water Resour. Res.* (2009).

[6] Belemtougri, P., Ducharne, A., Tazen, F., Oudin, L., Karambiri, H. Understanding key factors controlling the duration of river flow intermittency: Case of Burkina Faso in West Africa. *Axel J. Hydrol. Region. Stud*. 10.1016/j.ejrh.2021.100908 (2021).

[7] Vissin, E.. Impact of climatic variability and surface state dynamics on runoff in the Benin basin of the Niger River.* Hydrology*. https://theses.hal.science/file/index/docid/456097/filename/These_complete_Vissin.pdf (Université de Bourgogne, 2007, French: ffNNT: ff. fftel-00456097f).

[8] Bodjrènou, R. et al. Analysis of current and future climate variability and drought recurrence in Benin, West Africa. *J. Water Clim. Change* (2025).

[9] Guidigan, M. L. G. et al. Assessing land use/land cover dynamic and its impact in Benin Republic using land change model and CCI-LC products. *Earth Syst Environ* **3**, 127–137. 10.1007/s41748-018-0083-5 (2019).

[10] Dossou, J.F, Li, X., Kang, H. Boré, A.. Impact of climate change on the Oueme Basin in Benin. *Glob. Ecol. Conserv*. **28**, e01692. 10.1016/j.gecco.2021.e01692 (2021).

[11] Lawin, A. E., Hounguè, R., N’Tcha M’Po, Y., Hounguè, N. R. & Attogouinon, A. Afouda, AA Impacts of mid-century climate change on the flow of the Oueme River at the Bonou outlet (Benin). *Hydrology* **6**, 72. 10.3390/hydrology6030072 (2019).

[12] Biao, E.I. Assessing the impacts of climate change on river discharge dynamics in Oueme River basin (Benin, West Africa). *Hydrology* **4**(4). 10.3390/hydrology4040047 (2017).

[13] Cornelissen, T., Diekkrüger, B. & Giertz, S. A comparison of hydrological models for assessing the impact of land use and climate change on discharge in a tropical catchment. *J. Hydrol.* **498**, 221–236. 10.1016/j.jhydrol.2013.06.016 (2013).

[14] Kpegli, K.A.R., Alassane, A., Zouari, K., Ofterdinger, U. 2024. Delineation of a conceptual groundwater flow model of the Kandi basin in Benin (West Africa): Insights from isotopes, piezomETic and hydrological investigations. *J. Hydrol. Region. Stud.* **53**, 101804. 10.1016/j.ejrh.2024.101804 (2024).

[15] Djihouessi, M. B. & Aina, M. P. A review of habitat and biodiversity research in Lake Nokoué, Benin Republic: Current state of knowledge and prospects for further research. *Ecohydrol. Hydrobiol.* **19**(1), 131–145. 10.1016/j.ecohyd.2018.04.003 (2019).

[16] Bodjrènou, R. et al. Evaluation of reanalysis estimates of precipitation, radiation, and temperature over Benin (West Africa). *J. Appl. Meteorol. Climatol.* **62**, 1005–1022. 10.1175/JAMC-D-21-0222.1 (2023C).

[17] Bodjrènou, R., Sintondji, L. O. & Comandan, F. Hydrological modeling with physics-based models in the Oueme Basin: Issues and perspectives for simulation optimization. *Journal of Hydrology: Regional Studies* **48**, 101448. 10.1016/j.ejrh.2023.101448 (2023H).

[18] Bossa, Y. et al. Flood risk assessment in the lower valley of Oueme, Benin. *Open Journal of Modern Hydrology* **14**, 130–151. 10.4236/ojmh.2024.142008 (2024).

[19] Djihouessi, M. B. & Aina, M. P. A review of hydrodynamics and water quality of Lake Nokoué: Current state of knowledge and prospects for further research. *Region. Stud. Mar. Sci.* **18**, 57–67. 10.1016/j.rsma.2018.01.002 (2018).

[20] INSAE. *Population Numbers in Benin’s Villages and City Districts (RGPH-4, 2013)*. 3–83 (2013).

[21] Chikou, A. *Chapter 2 General Study Framework: The Oueme Delta*. https://orbi.uliege.be/bitstream/2268/315042/3/chap2.pdf (2006).

[22] Bodjrènou, R., Sintondji, L.O., M‘pon’tcha, Y. & Germain,D. Assessment of hydrologic data estimates from ERA5 reanalyses in Benin, West Africa. *Geosci. Data J*. **12**(1), e288. 10.1002/gdj3.288 (2025).

[23] Maxwell, R. M. & Miller, N. L. Development of a coupled land surface and groundwater model. *J. Hydrometeorol*. **6**(3), 233–247. 10.1175/JHM422.1 (2005).

[24] Richard, A. *Analyse du Cycle Hydrologique en Climat Soudanien au Bénin: Vers une Modélisation Couplée des Processus Latéraux et Verticaux* (Université Grenoble Alpes, 2014).

[25] Hector, B., Cohard, J.-M., Séguis, L., Galle, S. & Peugeot, C. Hydrological functioning of western African inland valleys explored with a critical zone model. *Hydrol. Earth Syst. Sci.* **22**, 5867–5888. 10.5194/hess-22-5867-2018 (2018).

[26] Herzog, A. et al. A paramETic sensitivity analysis for prioritizing regolith knowledge needs for modeling water transfers in the West African critical zone. *Vadose zone Juournal*10.1002/vzj2.20163 (2021).

[27] Cotillon, S. E. West Africa land use and land cover time series. In *US Geological Survey Fact Sheet Fact Sheet*. (2017).

[28] Cucchi, M. et al. WFDE5: Bias-adjusted ERA5 reanalysis data for impact studies. *Earth Syst. Sci. Data* **12**, 2097–2120. 10.5194/essd-12-2097-2020 (2020).

[29] Hersbach, H. et al. ERA5 hourly data on single levels from 1940 to present. In *Copernicus Climate Change Service (C3S) Climate Data Store (CDS)* (2023).

[30] Voldoire, A. et al. Evaluation of CMIP6 DECK experiments with CNRM-CM6-1. *Journal of Advances in Modeling Earth Systems* **11**(7), 2177–2213. 10.1029/2019MS001683 (2019).

[31] Bonnet R., Boucher O., Deshayes J., Gastineau G., Hourdin F., Mignot J., Servonnat J. & Swingedouw D. Presentation and evaluation of the IPSL-CM6A-LR ensemble of extended historical simulations. *J. Adv. Model. Earth Syst*. **13**, e2021MS002565. 10.1029/2021MS002565 (2021).

[32] Gupta, H. V., Kling, H., Yilmaz, K. K. & Guillermo, F. M. Decomposition of the mean squared error and NSE performance criteria: Implications for improving hydrological modelling. *J. Hydrol.* **377**, 80–91. 10.1016/j.jhydrol.2009.08.003 (2009).

[33] Le Lay, M., Saulnier, G.-M., Galle, S., Seguis, L., Métadier, M. & Peugeot, Ch. Model representation of the Sudanian hydrological processes: Application on the Donga catchment (Benin). *J. Hydrol*. **363**(1–4), 32–41. 10.1016/j.jhydrol.2008.09.006 (2008).

[34] Islam, M. D. et al. Factors affecting biopore-root interaction: A review. *Discov Agric* **2**, 67. 10.1007/s44279-024-00083-6 (2024).

[35] Shi, X., Qin, T., Yan, D., Tian, F. & Wang, H. A meta-analysis on effects of root development on soil hydraulic properties. *Geoderma* **403**, 115363. 10.1016/j.geoderma.2021.115363 (2021).

[36] Amou, M., Sambieni, K., Avossè, A.D., Bodjrènou, R. & Comandan, F. Short paper about the influence of climate variability on water availability in the Oueme Delta (Southern Benin). *Geo Eco Trop*. **46**(2), 343–348. https://geoecotrop.be/uploads/publications/pub_462_14.pdf (2022).

[37] Op de Hipt, F., Diekkrüger, B., Steup, G., Yira, Y., Hoffmann, T., Rode, M. & Näschen, K. Modeling the effect of land use and climate change on water resources and soil erosion in a tropical West African catch-ment (Dano, Burkina Faso) using SHETAN.* Sci. Total Environ*. **653**, 431–445. 10.1016/j.scitotenv.2018.10.351 (2019) (epub 2018 Oct 28). 10.1016/j.scitotenv.2018.10.351 30412888

[38] Ranjan, S P., So, K. & Sawamoto, M. *Effects of Climate and Land Use Changes on Groundwater Resources in Coastal Aquifers*. Vol. 7459. 2 (2016). 10.1016/j.jenvman.2005.08.008 16305816

[39] Yenehun, A., Nigate, F., Belay, A.S., Desta, M.T., Camp, M.V. & Walraevens, K. Groundwater recharge and water table response to changing conditions for aquifers at different physiography: The case of a semi-humid river catchment, northwestern highlands of Ethiopia. *Sci. Total Environ*. **748**, 1142243. 10.1016/j.scitotenv.2020.142243 (2020). 10.1016/j.scitotenv.2020.142243 33113708

[40] Höllermann, B., Giertz, S. & Diekkrüger, B. Benin 2025-Balancing future water availability and demand using the WEAP 'Water Evaluation and Planning’ system. *Water Resour. Manag*. **24**(13), 3591–3613. 10.1007/s11269-010-9622-z (2010).

[41] Etène, C., Dossou-Yovo, E., Sintondji, L. O. & Vissin, E. Impacts of climate change and vegetation cover dynamics on water resources in the Okpara Basin at the Kaboua outlet by 2025. *Journal de la Recherche Scientifique de l’Université de Lomé (Togo)* **4**, 129–144 (2017).

[42] Séguis L. et al. Contrasted land-surface processes along the West African rainfall gradient. *Atmos. Sci. Lett.* **1**, 31–37 (2011).

